# Polysubstance Use Patterns Among High Dose Benzodiazepine Users: A Latent Class Analysis and Differences Between Male and Female Use

**DOI:** 10.3389/fpsyt.2022.811130

**Published:** 2022-01-25

**Authors:** Lorenzo Zamboni, Igor Portoghese, Alessio Congiu, Thomas Zandonai, Rebecca Casari, Francesca Fusina, Anna Bertoldi, Fabio Lugoboni

**Affiliations:** ^1^Unit of Addiction Medicine, Department of Internal Medicine, Integrated University Hospital of Verona, Policlinico “G.B. Rossi”, Verona, Italy; ^2^Department of Neurosciences, University of Verona, Verona, Italy; ^3^Department of Medical Sciences and Public Health, University of Cagliari, Cagliari, Italy; ^4^Department of Sport Sciences, Sports Research Centre, Miguel Hernández University, Elche, Spain; ^5^Neuropharmacology on Pain and Functional Diversity (NED), Institute of Health and Biomedical Research of Alicante (ISABIAL), Alicante, Spain; ^6^Padova Neuroscience Center, University of Padova, Padova, Italy; ^7^Department of General Psychology, University of Padova, Padova, Italy

**Keywords:** benzodiazepine, addiction, latent class analysis, polyabusers, anxiety

## Abstract

Benzodiazepines (BZDs) represent one of the most widely used groups of pharmaceuticals, but if used for long periods of time they are associated with dependence and an increased risk of harmful effects. High-dose (HD) BZD dependence is a specific substance use disorder associated with a poor quality of life. It is especially important to pinpoint differences in HD BZD addict subgroups in order to tailor treatment to the individual's specific needs, also considering possible comorbidities with other substance use disorders. We conducted a study to evaluate HD BZD dependence (converted doses to diazepam equivalents, mg) in an Italian sample of 1,354 participants. We also investigated if and to which extent participants co-used other substances (alcohol, tobacco, cannabis/cannabinoids, cocaine, and heroin). We then performed latent class analysis (LCA) to identify the use patterns of these substances, finding three classes: participants in Class 1 (4.3% of the sample) had the highest probability of also using cocaine and alcohol (Polysubstance BZD users); Class 2 comprised subjects with the highest probability of being former heroin, cocaine, THC, and alcohol users (Former polysubstance BZD users); Class 3 represented mono-dependence BZD users (78.5% of the sample) and was the most prevalent among women, while young men were most prevalent in Class 1.

The present study underlines different characteristics in HD BZD users both concerning other addictions and sex, and also highlights the need for a stricter control of BZD use, ranging from prescriptions to sales.

## Introduction

Benzodiazepines (BZDs) are among the most commonly prescribed medications for insomnia and anxiety and are extensively used in clinical practice. BZDs act as positive allosteric modulators of the GABA-A (Gamma-Aminobutyric Acid Type A) receptor (1). A number of studies have evidenced that benzodiazepines should be considered a suitable treatment for specific clinical situations and for short-term use only (2–4): indeed, long-term use of this class of drugs increases the likelihood of adverse effects and dependence and should therefore be implemented with caution. Alternative short or intermittent treatments could have important benefits for patients and should be taken into consideration when deciding on a specific course of treatment (5).

Long-term BZD users range from 6 to 76% of total users. Fifteen to forty four percent of them present moderate-to-severe withdrawal symptoms, and 3–4% show a dependence (6).

High-dose (HD) BZD dependence is considered a specific substance use disorder (7) and it consistently reduces quality of life in patients that suffer from it (8, 9). Ohayon and Lader (10) conducted a cross-sectional survey in various European countries (France, Germany, and Italy) and in the UK, and found that an estimated 0.14% of the general population took higher-than-recommended doses of anxiolytic medications, while 0.06% reportedly abused hypnotics (10). These numbers are in line with the 0.16% of high-dose BZD users reported in Switzerland (11) and point toward HD BZD abusers being around 1.5 million in Europe and 600,000 in the United States.

Long-term BZD use was reported to be associated with abnormalities in cognitive functions, including attention, memory and learning, a higher risk of delirium, cognitive decline and accidents (12–21).

BZD withdrawal in patients is an especially discomforting experience. To alleviate withdrawal symptoms, therapeutic strategies such as gradual tapering of the dosage or substituting the target BZD with an equivalent dose of another long-acting benzodiazepine and then tapering have been developed (22, 23).

BZDs have been reported to be secondary drugs of abuse for most individuals, and a much smaller number report BZDs as the primary drugs of abuse. BZD abuse is mainly associated with opioids (54.2%) and alcohol (24.7%) abuse. As reported by Schmitz (24) in her recent review, about 1 in 5 people who abuse alcohol also abuse benzodiazepines (25).

In the last decade, research has been increasingly focused on patterns of polyabuse defined as the use of more than one drug during a specific time period. While patterns of use and characteristics of polyabusers have been examined among many substance users (such as alcohol, cocaine, heroin, etc.), not much is known about polyabuse patterns among BZD abusers. Several studies have aimed to address this complexity by identifying homogenous subgroups of patients who have similar outcomes (26, 27). The same pre-existent characteristics of the patients, indeed, do not consistently produce the same effects.

Understanding the distribution and determinants of polysubstance use is crucial for planning overdose prevention programs and policies. The problem of polydrug use has also been acknowledged as crucial in the context of treatment. There is general consensus that the effects of combining multiple substances of abuse are often problematic to predict and can increase the risks of accidents, overdose and death (28). In this respect, the last 15 years have seen an increased focus on person-centered methodologies that statistically uncover subpopulations with distinct combinations of polysubstance use (29, 30). Latent Class Analysis (LCA) is a type of finite mixture model that is used to identify and describe homogenous subgroups within a heterogeneous population based on the similarity of their response patterns (31). This method has been widely used in previous studies to examine substance use patterns (32–35).

LCA has been used in other studies to identify subgroups of substance use, misuse and addiction, e.g. to tobacco, internet etc. (36–40); notably, it has been used to differentiate problematic alcohol users from addicted users (41).

Regarding BZD addiction, LCA has been utilized by Votaw et al. (42) in a general population sample. In their study, the authors identified three distinct latent classes: limited polysubstance use class, binge alcohol and cannabis use class, and opioid use class.

However, to the best of our knowledge, the present study is the first to examine polysubstance use patterns among individuals who use BZDs as their primary drugs of choice in order to identify different classes of BZD users characterized by distinct substance combinations emerging from LCA.

## Methods

### Participants

All participants were in treatment for BZD detoxification at the Addiction Unit of the Verona University Hospital in Verona (Italy). During the study period (November 2003 to February 2020) 1,354 people were screened at the Addiction Unit of the Department of Internal Medicine at the Verona University Hospital for high-dose BZD dependence. Inclusion criteria were: being over 18 years of age; meeting the DSM-IV (43) criteria for benzodiazepine dependence, with more than 6 months' use; high dose benzodiazepines use (HDU). The DSM-IV criteria was applied by the clinician.

All patients also had to have so-called problematic use, defined by either mixing BZDs, escalating dosage, and/or using BZDs for recreational purposes (8, 9, 44). In accordance with previous literature (45, 46), the proposed detoxification program they enlisted in consisted in a 7-day continuous slow infusion of flumazenil (FLU-SI) in an inpatient setting, followed by interventions such as counseling, cognitive-behavioral therapy, and pharmacological therapy to prevent BZD relapse.

Patient demographics, the type of BZD they used and what it had been prescribed for, the duration of its use and its mean daily dose in the previous 3 months, its preferred administration route, comorbid abuse of other substances or other psychiatric disorders and detoxification attempts were assessed upon admission to our Unit.

The definition of what constitutes a “high dose” is still controversial and no real consensus exists about the appropriate clinical criteria that should be applied; in our study, we recommended inpatient treatment if a patient's BZD intake was at least 5 times higher than the maximum defined daily dose (DDD). Among the BZDs considered, we also included so-called Z-drugs. BZD use was quantified as standardized as diazepam dose equivalents.

The study was conducted according to the Declaration of Helsinki. Its protocol was approved by the ethics committee of the Verona University Hospital (approval code 683CESC) and fully adhered to its guidelines. Patients and controls gave written informed consent to participate in the study and to receive off-label administration of flumazenil (patients only).

### Measures

Participants were asked questions regarding their demographic profile, including sex, age, age of first use, education, marital status, and employment status. BZD dependence duration was considered by converting doses to diazepam equivalents (DDDE, mg) (47) and calculating the mean diazepam dose/day.

Furthermore, participants' history of drug addiction and simultaneous drug use was assessed, considering alcohol, tobacco, cannabis/cannabinoids, amphetamines/methamphetamines, barbiturates/sedatives, cocaine, and heroin. To better quantify these variables, we assigned the following: (0) no drugs/alcohol used in the past 12 months, (1) previous history of drug/alcohol addiction, (2) addicted.

Information on these variables was mainly obtained from medical records. DDDE data were based on self-report.

### Data Analysis

LCA was implemented to identify the use patterns of seven substances (other than BZDs): alcohol, tobacco, cannabis/cannabinoids, cocaine, and heroin. As the usage rate of amphetamines/methamphetamines and barbiturates/sedatives was low, we removed them from the analyses. LCA including one to six latent classes was estimated by employing the robust maximum-likelihood estimator (MLR) MPlus 7. The LCA was conducted by using 5,000 random sets of start values and 1,000 iterations, and the 500 best solutions were retained for final stage optimization (48, 49). In deciding how many classes should be retained, we considered the statistical appropriateness and consistency with respect to the theoretical meaning and conformity of the extracted classes (50–53).

Different information criteria (IC)-based fit statistics were examined in selecting numbers of classes. ICs follow the principle of parsimony controlling for overfitting and providing a standardized way to balance sensitivity and specificity (54). The following ICs were considered: the Bayesian Information Criterion [BIC; (55)], the Akaike Information Criterion [AIC; (56)], the Constant AIC (CAIC), the Sample Adjusted Bayesian information criterion (SABIC), and the bootstrapped likelihood ratio [BLRT; (57)]. The BLRT test compares the improvement between K-class model with a K-1 class model, providing *p*-values that can be used to justify the inclusion of one more class. Finally, we examined the accuracy with which models classify individuals into their most likely class by considering the entropy of each model. Entropy values range from 0 to 1 and indicate the clarity of class specification, with scores closer to 1 indicating better fit of the data into the prescribed class structure. According to the recommended fit indices (52), the optimal class solution would have the lowest BIC values, lowest AIC values, lowest CAIC values, lowest SABIC values, a significant BLRT *p* value, relatively higher entropy values, and conceptual and interpretive meaning. Furthermore, when comparing a K-class model with a K-1 class model, a significant BLRT test indicates that the model with K classes is optimal.

Furthermore, information criteria were depicted through “elbow plots” showing the improvements related with additional classes (53). More specifically, the optimal number of classes should be the value at which the slope flattens, plus and minus a class.

Then, we analyzed the associations between the identified classes and the sociodemographic variables of the participants. In this sense, the consideration of predictors should not qualitatively change the classes (49). More specifically, we regressed the latent classes on age, age of first use, sex, and employment (yes/no) in a series of multinomial logistic regressions. The R3STEP method in MPlus (58–60) was used.

## Results

### Participant Characteristics

A cross-sectional survey study was carried out. From the starting 1,354 questionnaires, 265 were removed because of missing responses (> 5%) about relevant variables to this study. The final sample comprised 1,088 subjects.

As shown in Table 1, slightly more than half (51%) were female and the mean age was 45.85 (SD ± 10.82) years. Regarding employment, 53.5% were employed. Characteristics of the sample are summarized in Table 1.

**Table 1:** Demographic characteristics of the patients according to the type of high-dose.

		**n**	**%**	**M**	**SD**
Sex	Male	534	49.1%		
	Female	554	50.9%		
Age (years)				45.85	10.82
Employment	yes	582	53.5%		
	no	506	46.5%		
Age of first BZD use (years)				30.60	10.68
Continuous use of BZD (months)				92.97	88.34
**Reason for BZD use**					
Anxiety	yes	347	31.9%		
	no	741	68.1%		
Panic attacks	yes	77	7.1%		
	no	1,011	92.9%		
Insomnia	yes	617	56.7%		
	no	471	43.3%		
Drug-seeking behavior	yes	137	12.6%		
	no	951	87.4%		
other reasons	yes	96	8.8%		
	no	992	91.2%		
Heroin	no	923	84.8%		
	former	133	12.2%		
	yes	32	2.9%		
Cocaine	no	810	74.4%		
	former	216	19.9%		
	yes	62	5.7%		
THC	no	862	79.2%		
	former	184	16.9%		
	yes	42	3.9%		
ALCOHOL	no	747	68.7%		
	former	193	17.7%		
	yes	148	13.6%		
DDDE (mg)				382	483

*BZD, Benzodiazepine; DDDE, diazepam equivalents; M, mean; SD, standard deviation; THC, tetrahydrocannabinol; BZD, benzodiazepines and polydrug misuse*.

### Latent Class Analysis

Fit indices resulting from the latent profile models containing up to 6 classes are provided in Table 2.

**Table 2:** Fit indices for LCA models with 1–5 classes.

**Model**	**LL**	**#fp**	**Scaling**	**AIC**	**CAIC**	**BIC**	**SABIC**	**Entropy**	**BLRT**
1 Class	−2884.35	8	1.000	5784.69	5832.63	5824.63	5799.22		Na
2 Classes	−2496.35	17	1.015	5026.70	5128.56	5111.56	5057.57	0.863	<0.001
3 Classes	−2482.87	26	1.070	5017.74	5173.53	5147.53	5064.95	0.861	<0.001
4 Classes	−2475.28	35	1.038	5020.57	5230.29	5195.29	5084.12	0.917	ns
5 Classes	−2469.85	44	1.040	5027.70	5291.36	5247.36	5107.60	0.881	ns
6 Classes	−2465.77	53	1.000	5037.54	5355.12	5302.12	5133.78	0.919	ns

*AIC, Akaike information criterion; CAIC, Constant AIC; BIC,Bayesian Information Criterion; SABIC, Sample adjusted BIC; BLRT, bootstrap likelihood ratio test; LL, log-likelihood; #fp, number of free parameters*.

**Figure 1:**
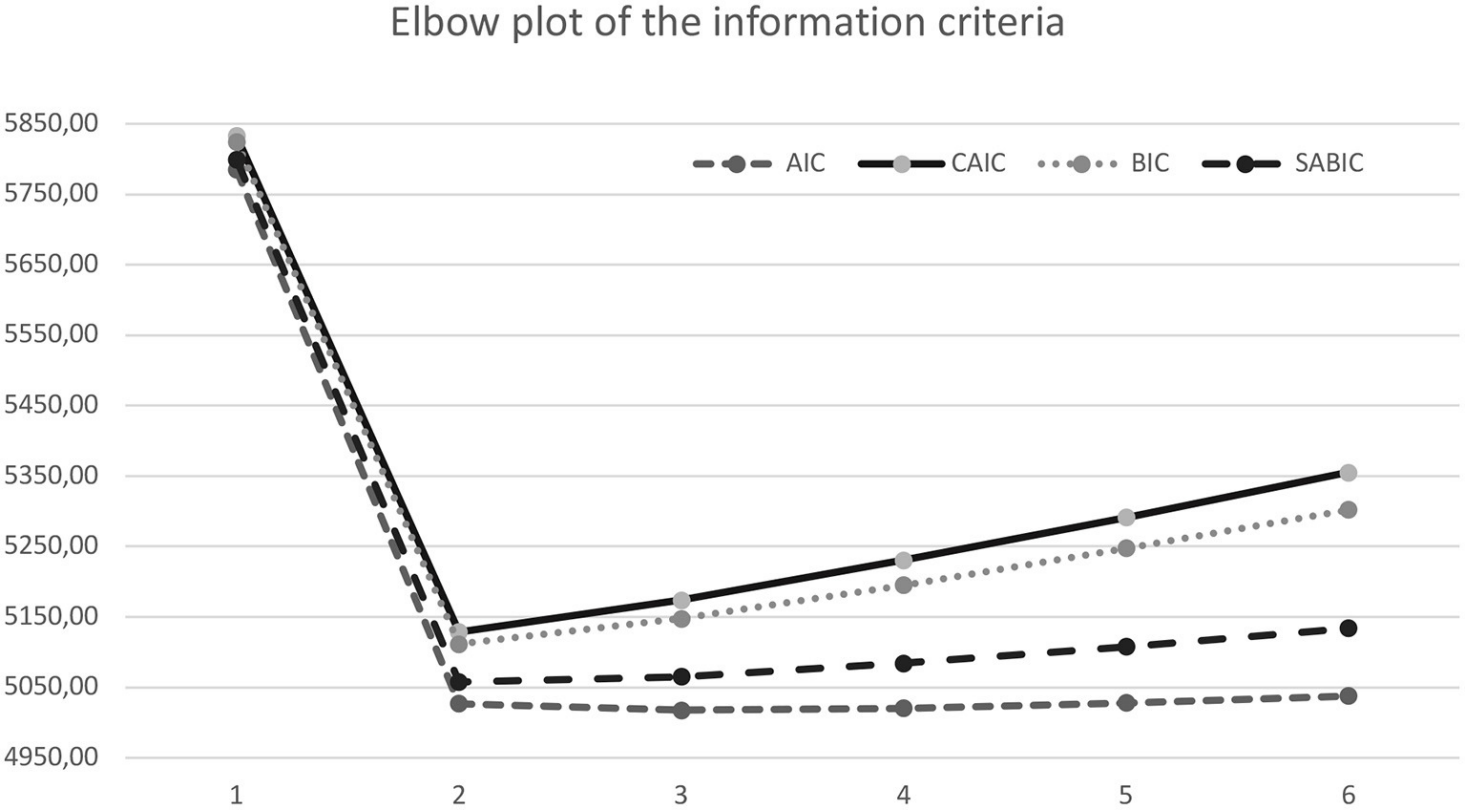
Elbow plot of the information criteria.

Taken as a whole, the 2-, and 3- class solutions showed the better fit as they were supported by the BIC and ABIC values, and BLRT tests (Table 2). Then, we compared the 2-, and 3- class solutions. Comparisons of the AIC, BIC, and ABIC values for all the models were contrasted in an elbow plot (Figure 1). Nylund et al. (52) suggest that lower BIC, AIC, CAIC and ABIC values indicate a better fit in class selection. However, for both 2- and 3- class solutions those values did not differ greatly across models. In addition, although BLRT distinguishes between class models (52), BLRT significance values did not differ across the two solutions, so we examined both options considering whether classes were theoretically meaningful and interpretable. We inspected the proportion of participants in each class finding that, concerning the 3-class solution, the smallest class drastically dropped to <5%. Although the principle of parsimony is generally to be followed, in our case adding a third class resulted in the addition of a well-defined, qualitatively distinct and theoretically meaningful class. Thus, the interpretability and clinical utility of the 3-class model was superior. This solution provided a reasonable level of classification accuracy, with an entropy value of 0.861. These results clearly suggest the high level of classification accuracy of these solutions, with average posterior probabilities of class membership varying from 0.69 to 0.96 (*M* = 0.85), with low cross-probabilities, ranging from 0.014–0.189 (*M* = 0.072).

The retained 3-class solution is represented in Table 2. Class 1 represents 4.3% of the sample (*n* = 47, latent class membership probability = 0.69) and participants in this class had the highest probabilities of using cocaine (53%), and alcohol (56%). Thus, this class was labeled as Polysubstance BZD users. Class 2 represents 17.2% of the sample (*n* = 186, latent class membership probability = 0.91) and participants in this class had the highest probabilities of being former heroin (62%), cocaine (77%), THC (66%), and alcohol users (36%). Thus, this class was labeled as Former polysubstance BZD users. Finally, class 3 represents 78.5% of the sample (*n* = 855, latent class membership probability = 0.96) and participants in this class had the highest probabilities of not using heroin (99%), cocaine (94%), THC (95%), and alcohol (77%). Thus, this class was labeled as mono-dependence BZD users.

Table 3 shows the sociodemographic predictors of the LC membership, including sex, age, age of first use, and employment. We performed a series of logistic regression analyses (R3STEP) where the categorical latent class variable was regressed on sex (0 = male; 1 = female), employment (yes/no), and the continuous age variables (Table 4). This analysis showed that males were more likely than females to be in class 1 (Polysubstance BZD users; OR = 17.83), and class 2 (Former polysubstance BZD users OR = 7.69) compared to class 3 (BZD users). Younger individuals were more likely to be in class 1 (Polysubstance BZD users; OR = 1.13) and class 2 (Former polysubstance BZD users OR = 1.09) compared to class 3. Finally, employed individuals were less likely to be in class 2 (Former polysubstance BZD users OR = 1.62) compared to class 3 (BZD users). Concerning the age of first use, no statistical differences were found among the three classes.

**Table 3:** Characteristics of drugs used and socio-demographics, stratified by latent class.

		**Class 1**		**Class 2**		**Class 3**	
		**N**	**M (SD)**	**N**	**M (SD)**	**N**	**M (SD)**
Sex	Male	38		143		353	
	Female	9		43		502	
Age			38.04 (8.53)		40.46 (7.92)		47.45 (10.92)
Employment	yes	27		80		475	
	no	20		106		380	
Age of first BZD use			25.89 (10.05)		27.85 (9.69)		31.46 (10.76)
DDDE (mg)			416 (432)		412 (474)		373 (488)

*BZD, Benzodiazepine; DDDE, diazepam equivalents; M, mean; SD, standard deviation*.

**Table 4:** Odds coefficients for the 3-class model with sex, age, age first use, and employment as covariates.

	**Class 1 vs. 2**		**Class 1 vs. 3**		**Class 2 vs. 3**	
	**Estimate (S.E.)**	**OR**	**Estimate (S.E.)**	**OR**	**Estimate (S.E.)**	**OR**
Sex (Male)	0.84 (1.37)	2.32	2.88 (1.30)^*^	17.83	−2.04 (0.28)^*^	7.69
Age	0.03 (0.04)	1.03	−0.12 (0.04)^**^	1.13	−0.09 (0.01)^*^	1.09
Age first use	0.26 (0.65)	1.30	−0.08 (0.07)	1.08	0.004 (0.015)	1.00
Employement (Yes)	0.08 (0.75)	1.08	0.22 (0.63)	1.25	0.48 (0.14)^*^	1.62

*OR, Odd Ratio; S.E., standard error; Class 1, Polysubstance BZD users (n = 47); Class 2, Former polysubstance BZD users (n = 186); Class 3, BZD solo users (n = 855)*.

* *p < 0.05*,

** *p < 0.01*.

## Discussion

Over the past 40 years, BZD dependence has been on the rise as a public health concern around the world (61). The present study aims to examine patterns of polysubstance use among a sample of Italian adults with BZD dependence.

Given its clinical relevance, we aimed to disentangle the patterns of polysubstance use among a sample of Italian adults that were also misusing BZDs. Our findings revealed three main types of BZD-HD users: of these, the vast majority only abused BZDs (78.5% of the sample), while the other two groups were either more likely polydrug users or former polydrug users. These results highlight that, while the overlap between BZD use and the abuse of other substances is well-documented, a large portion of BZD users are actually less likely to also be using heroin, cocaine and alcohol, as was also reported in other studies (42, 61).

However, the percentage of users who showed a high probability of polyabuse or of being former polyabusers is far from negligible. The simultaneous use of BZDs and other substances is especially concerning given that it may increase the risk of overdose (25), and these two classes of subjects present high treatment complexity.

It is important to note that biological, psychological and social factors influence personal prognoses and treatment responses (62, 63). Many adverse consequences are associated with polydrug abuse, such as an increased fatal and non-fatal overdose (64, 65), self-harm (66), infectious disease (67, 68), risky sexual behavior/risky injection practices (68, 69), criminal involvement (70–72), suicidal ideation/attempt (73, 74), violence and reckless driving (39, 75), mental and physical impairment (75, 76) and social dysfunction (77).

However, the association between these risks and polyabuse (also including BZDs) is not completely clear. Some studies point toward BZD dependence increasing them by markedly increasing disinhibition (73, 74).

Another important aspect is the fact that BZD dependence with greater psychological severity also have poorer treatment outcomes (73, 74, 78).

The most frequent type of patients that we treat in our Addiction Unit is assignable to Class 3, which has the highest probability of not using any substance other than high doses of BZDs. For these patients, treatment with FLU-SI was shown to be efficacious (7).

There seems to be a gender gap concerning the prescription of psychoactive drugs, with BZDs more frequently prescribed to women (79, 80). This study suggests differences in the psychopathology underlying high-dose BZD use: on one hand males tend toward polydrug use, and on the other hand BZD-only users are for the most part female. The latter scenario might be due to several causes, including females being more prone toward anxiety and mood disorders requiring medication, and the tendency to prescribe BZDs more to women (81, 82). Women, in order to soothe psychological distress, tend to call on medical attention more than men, who more frequently resort to other means outside the healthcare system, e.g. alcohol use (83).

The third class is the most numerous among women, also suggesting that BZD addiction could be a cross phenomenon, not only concerning subjects with a history of polyaddictions. This is interesting, because our results suggests that BZD addiction could be a problem that involves a large segment of the general population.

Concerning both age and sex, we found that young men had a higher probability of being included in classes 1 (Poly-substance BZD users) and 2 (Former polysubstance BZD users). Several studies in the general population support the fact that the male sex and a younger age are associated with binge alcohol and cannabis use (33, 84). Women with cocaine and heroin addiction seem less likely than men to develop a comorbidity to alcohol (85). Since the 1980s, studies on heroin and cocaine users have indicated that women present a shorter-lasting addiction than men, and they enter in treatment at a younger age (86, 87). Westermeyer and Boedicker (88), regarding the abuse of multiple substances and their respective treatment, indicated that women progressed more quickly from drug use to dependence: that is, women used each drug (except cocaine) for a shorter period, while rates of dependence remained constant. Moreover, women entering treatment exhibited a more severe clinical profile due to the greater consequences of drug use/abuse in women relative to men (85, 89–91).

The present analysis had several methodological limitations. First, data from the present study are cross-sectional, therefore we cannot make causal conclusions about findings. Second, data were from retrospective, self-report measures. There is the risk that substance use could be underreported when comparing self-report measures with biological markers (92). In fact, despite the self-report measures finding a wide use in the context of substance abuse problems (93, 94), their use is still a matter of debate, due to the limitations related to their use, such as the patients' desire to show a positive self-image or difficulties in remembering consumption episodes and dosages taken (95). Third, information on lifetime or past year use was not available for all substances except BZDs. Fourth, actually there is no a clear definition of high dose of BZD. Finally, we were unable to examine subgroup differences between types of non-medical prescription BZD use.

## Conclusion

The present study underlines three different classes of BZD high dose abusers. The third class is the most represented and presents a mono-addiction (high dose BZD addiction). Our results and clinical experience highlight the need for a stricter control of BZD use, ranging from prescriptions to sales. While other BZD abuser studies show a female prevalence, our sample was more balanced regarding sex, but this is the first study with this peculiarity. This study also underlines the potential of LCA in improving knowledge of BZD abusers. Since LCA identifies homogeneous subgroups, this division could be used to plan and choose different and specific treatments. Further studies with LCA could be crucial especially in the field of BZD addiction, which would greatly benefit from more detailed studies.

## Data Availability Statement

The raw data supporting the conclusions of this article will be made available by the authors, without undue reservation.

## Ethics Statement

The studies involving human participants were reviewed and approved by 2822CESC. The patients/participants provided their written informed consent to participate in this study.

## Author Contributions

FL and RC were responsible for the study concept and design. IP, RC, and LZ contributed to the data acquisition. IP assisted with the data analysis and interpretation of findings. LZ, IP, FF, TZ, AB, and AC drafted the manuscript. All authors critically reviewed the content and approved the final version of the manuscript for publication.

## Conflict of Interest

The authors declare that the research was conducted in the absence of any commercial or financial relationships that could be construed as a potential conflict of interest.

## Publisher's Note

All claims expressed in this article are solely those of the authors and do not necessarily represent those of their affiliated organizations, or those of the publisher, the editors and the reviewers. Any product that may be evaluated in this article, or claim that may be made by its manufacturer, is not guaranteed or endorsed by the publisher.
